# Research on multi-effect evaporation salt prediction based on feature extraction

**DOI:** 10.1038/s41598-020-75112-7

**Published:** 2020-10-22

**Authors:** Bo-Lun Chen, Yong Hua, Guo-Chang Zhu, Min Ji, Hong-Fei Zhu, Yong-Tao Yu

**Affiliations:** School of Computer and Software Engineering, Huaiyin Institute of Technology, Huaian, 223003 China

**Keywords:** Chemistry, Mathematics and computing, Physics

## Abstract

In the multi-effect evaporation salt making process, the smooth operation of the salt making process is crucial. As the salt production process continues, many unstable factors will cause the salt production process not to proceed smoothly. These factors can be discovered in advance by predicting the salt production data, thus, it is of great significance to predict the multi-effect evaporation salt production data. In the process of multi-effect evaporation and salt production, the multiple salt-making devices make the influence between the parameters closer, and the influence of a single parameter on itself is sometimes ductile. Therefore, the data of multi-effect evaporation and salt production have the characteristics of high dimensions, high complexity and temporal information. If the historical salt production data is used for data prediction directly, the prediction model will take a long time and the prediction effect is not good. Thus, how to predict the multi-effect evaporation salt production data is the main research problem of this paper. In view of the above problems, according to the characteristics of multi-effect evaporation salt production data, this paper analyzes and improves the self encoder for feature extraction of multi effect-evaporation salt production data, so as to solve the problem of high dimensions and high complexity of salt production data. On this basis, combined with the time-series information contained in the salt production data, a multi-effect evaporation salt production data prediction model is proposed based on long-term and short-term memory cycle neural network to solve the prediction problem of time-series salt production data. Experiments show that the prediction model can predict and prevent the problems in salt production line in advance. It has a certain theoretical research value and application value in the intelligent production process and production line optimization of salt chemical industry.

## Introduction

The salt chemical industry is an important part of the chemical industry and an important source of the economy. With the continuous development of the chemical industry, the salt chemical industry is faced with the challenge of developing from a highly labor-intensive and high-energy production method to a low-energy and high-efficiency direction. In the face of this challenge, how to optimize the salt production process is the problem faced by the whole salt chemical industry. At present, the research on neural network, artificial intelligence, and big data is in-depth. How to apply them to the optimization of salt production process and get a better solution is particularly important. In recent years, the construction of industrial information infrastructure has been gradually accelerated, and the chemical production process has gradually completed the information coverage. The integration of emerging computer technology and industrial development provides the basis for the application of intelligent technology in the industrial field. The salt chemical industry is no exception. Most salt-making factories have supporting distributed control systems. DCS(Distributed Control System) uses computer technology to centrally control and manage the production process, and this system will support a background database to record a large amount of historical production data. These historical production data are of great value for the solution of optimizing the salt production process through neural network technology. Salt chemical companies mostly use mature multi-effect evaporation and combined heat and power technology to produce salt. Although the salt production technology is relatively mature, after years of production, the problems that occur in the production process will gradually accumulate.

In the process of salt production, although the process of salt production is monitored by the DCS, when the staff observe the problem data, the problems in the production line have already occurred. For example, the multi-effect evaporator will have the problem of scaling at the salt discharge foot during the salt making process. The main reason is that the washing water is introduced into the salt discharge foot of the evaporation tank, and the solution at the salt discharge foot has a more violent cooling process, especially the 1-3 effect evaporation tank. Therefore, it is particularly important to predict the changing trends of the key parameters.

In the process of multi-effect evaporation, the production process is continuous, and there is a potential connection and influence between different production parameters. Therefore, similar scaling problems can be obtained by analyzing the historical production data and the potential correlation between the production parameters, thus predicting the trend of key parameters in salt production, adjusting the salt production parameters in advance, and preventing problems. Therefore, it is of great practical and economic significance to establish a parameter prediction model based on the historical salt production data and predict the key salt production parameters through neural network technology. In the actual multi-effect evaporation salt production process, the salt production line will generate a large number of production data with high dimension and nonlinearity in real time. Generally speaking, if the production data with high dimension and large nonlinearity is directly used to train the prediction model, it will not only make the prediction model training efficiency low, but also make the prediction accuracy relatively low. Therefore, how to process the original salt production data and train the prediction model becomes the key point of the prediction problem. Compared with low-dimensional data, high-dimensional data contain more complex information and data analysis is more difficult. Using the high-dimensional data to train the prediction model directly will lead to the problems of long training time and poor prediction accuracy. Feature extraction is the key technology to deal with high-dimensional data. A series of transformations are carried out to map high-dimensional data into low-dimensional subspace, so as to obtain the low-dimensional feature representation of the original data. Using the extracted feature data of the original data to train the prediction model will greatly reduce the training time of the prediction model and improve the performance of the prediction model.

The goal of various learning algorithms is to complete the prediction of the data, thus data prediction is the key problem that the learning algorithm needs to solve. The principle of data prediction is to learn the fixed pattern of the original data through the learning model, thereby predicting the data, thus data prediction is the ultimate goal of the entire learning process. There are many algorithms for data prediction in the computer field, such as recurrent neural networks. RNN (Recurrent neural network) is a type of neural network with memory function and is suitable for learning time-series data. RNN is the same as the general neural network, which is composed of explicit layer neurons, hidden layer neurons, and output layer. The difference is that hidden layer neurons are not only affected by the input neurons, but also affected by the current state of the hidden layer neurons themselves. The subsequent state, so RNN has the memory ability, which has an advantage for the learning task of time-series data. Power system load forecasting, especially the short-term power load forecasting of individual users, plays an important role in future grid planning and operation. In addition to large-scale centralized residential power supply, the power load of a single user involves high volatility and uncertainty, thus the prediction of power load is quite challenging^1^. Kong et al. proposed a framework based on recurrent neural networks to solve the above problems and tested it on public data. The proposed method outperformed other algorithms in the short-term load forecasting of individual users. The time sequence of power load is highly nonlinear, thus it is very difficult to accurately predict the power load. Zheng et al. found that short-term power load prediction can predict the future short-term load, and proposed a prediction framework based on long-short-term memory recurrent neural network. This framework can make use of the long-term dependence in the electrical load time series for accurate prediction^2^. Wang et al. studied the prediction problem of helping information spread on graphs through representation learning, especially the probability of inactive nodes activated at the next time point in the cascade of information dissemination^3^. The author believed that the deep learning method is successful in diffusion prediction, but it is not enough to explore the cascade structure, thus the author proposed that cascade is not only a sequence of nodes sorted by activation time, it has a richer potential structure, indicating the diffusion process on the data graph. The author introduced a new data model, the diffusion topology, to fully describe the cascade structure. The author found through research that using existing neural networks to model diffusion topology is a difficult task. Therefore, a novel topological recurrent neural network based on recurrent neural network was proposed, and it showed promising performance on multiple real data sets. For the personalized recommendation problem, Donkers et al. used recurrent neural networks to model the sequence data and generate effective personalized recommendations^4^. Sherstinsky et al. aimed at the current problem of insufficient RNN training formulas^5^, drawing on signal processing theory, drawing standardized RNN formulas from differential equations, and proposing and proving a precise statement that produced RNN expansion techniques, which helped to understand RNN more clearly. For the problem of traffic speed prediction, Lv et al. proposed a model based on recurrent neural network to achieve more accurate traffic speed prediction^6^. The author learns time-series patterns by integrating RNN and convolutional neural network models to adapt to the traffic dynamics in the surrounding area. For the problem of how to extract useful information from protein sequences, Liu et al. used RNN for protein function classification^7^.

The multi-effect evaporation salt production process is a continuous production process, thus the salt production data have the characteristics of time series. Time-series data are a type of data with a certain order in the time series^8^, that is, the data change continuously according to the time axis, such as weather and air quality data, video data, stock data and vehicle flow data^9^, etc. They are widely used in many fields^10^. The salt production data generated by the multi-effect evaporation salt production process is a kind of time-series data, thus this paper studies the time-series data prediction algorithm.

In this paper, the principle and construction of the feature extraction model based on neural network are studied, and the feature extraction model is used to extract the feature of the multi-effect evaporation salt production data, then the feature extracted from the original production data is used to train the prediction model, and then the key data of the multi-effect evaporation salt production are predicted.

## Results

### Multi-effect evaporation salt production data

This article mainly conducts prediction research on multi-effect evaporation salt production data. The salt production data used are all derived from real multi-effect evaporation salt production data. The salt production data are collected by DCS and stored in the background database, with one record per minute Data, the data contains 1935 parameter values. Among them, 589 parameters are numeric parameters, and the remaining parameters are non-numeric parameters. In this article, only numeric parameters are used. In terms of the collection of experimental data, a total of 80,000 consecutive time periods of data were obtained as experimental data sets, each of which contained 589 numerical parameters. Because this article is a prediction study on salt production data, some key parameters in the salt production line are selected as the prediction targets, namely the solid–liquid ratio, vapor pressure (kPa) and the vapor pressure (kPa) in the four evaporation tanks EV11 to 14(i.e., the actual ID of evaporation tank in the factory) in the salt production line. Salt leg flow ($m^3/h$), where the attributes of key parameters are shown in Table 1.

**Table 1:** Autoencoder training algorithm ae_train.

Parameter	Max	Min	Medium	Mean	Variance	Standard
Solid–liquid ratio in EV11	57.38	0.00	11.25	11.48	18.07	4.25
Solid–liquid ratio in EV12	60.00	0.00	15.16	15.34	18.91	4.35
Solid–liquid ratio in EV13	60.00	0.00	14.01	14.38	20.85	4.57
Solid–liquid ratio in EV14	60.00	0.00	18.73	17.50	137.7	11.74
Steam pressure in EV11	38.02	− 74.11	− 9.16	− 8.97	86.30	9.29
Steam pressure in EV12	− 33.30	− 83.22	− 55.09	− 54.85	18.95	4.35
Steam pressure in EV13	− 74.80	− 89.12	− 80.33	− 80.33	2.14	1.46
Steam pressure in EV14	− 93.00	− 96.12	− 94.62	− 94.62	0.13	0.36
Flow in EV11	90.02	0.00	39.16	37.80	144.06	12.00
Flow in EV12	66.11	0.00	33.15	32.15	122.73	11.08
Flow in EV13	80.02	0.00	27.75	21.45	244.98	15.65
Flow in EV14	130.03	0.00	61.27	59.26	536.35	23.16

Table 1 shows the maximum, minimum, median, mean, variance and standard deviation of the parameters. According to the variance and standard deviation, it can be found that the fluctuations of the parameters of EV14 solid–liquid ratio, EV11 steam pressure and EV11 to 14 salt leg flow rate are very sharp, thus the degree of nonlinearity is extremely high, which are difficult parameters to predict. The variance and standard deviation of EV13 steam pressure and EV14 steam pressure are small, and the data change is relatively stable, thus they belong to better predicted parameters. Generally speaking, the experiments in this paper include more difficulty to predict parameters. Because the unit of different parameters of the original salt-making data is different, that is, the dimensions are different, it cannot be directly used for model training. Therefore, this paper preprocesses the experimental data, that is, the dimensions of the experimental data set are converted to be the same through a standardized method. The standardization method used in this article is MinMaxScaler standardization, as shown in formula (1). Among them, min is the minimum value of the parameter, and max is the maximum value of the parameter. In this way, all parameters in the experimental data can be mapped to the scope between 0 and 1, so that the standardized experimental data have the same dimension.

1 $$\begin{aligned} x=\frac{x-min}{max-min} \end{aligned}$$

After standardization, this paper divides the experimental data. In summary, there are a total of 80,000 experimental data, each of which contains 589 parameter values. In this paper, the first 70,000 data are used as the training set for the training of the feature extraction models and prediction models, and thereafter 10,000 data are used as the test set used for the evaluation of the model prediction performance.

### Experiment preparation

The experiment is divided into 3 parts in total: training of the feature extraction model based on deep confidence network, training of the AE+LSTM(i.e., Autoencoder combine with Long short-term memory) model and prediction analysis of the AE+LSTM model. The Autoencoder is composed of two modules: an encoder and a decoder. In this paper, the encoder is composed of one explicit layer and 2 hidden layers, and the decoder is composed of 2 hidden layers. In this paper, the number of neurons in the explicit layer of the encoder is set to 589, the number of neurons in the first hidden layer and the second hidden layer of the encoder is set to 200 and 50, and the number of neurons in the first hidden layer and the second layer of the decoder is set to 200 and 589. The initial values of the edge weights in the Autoencoder network are randomly generated by a uniform distribution function, and the bias on each neuron is initialized as 0. The setting of the LSTM model has been introduced in the third.

### Feature extraction model training

**Figure 1:**
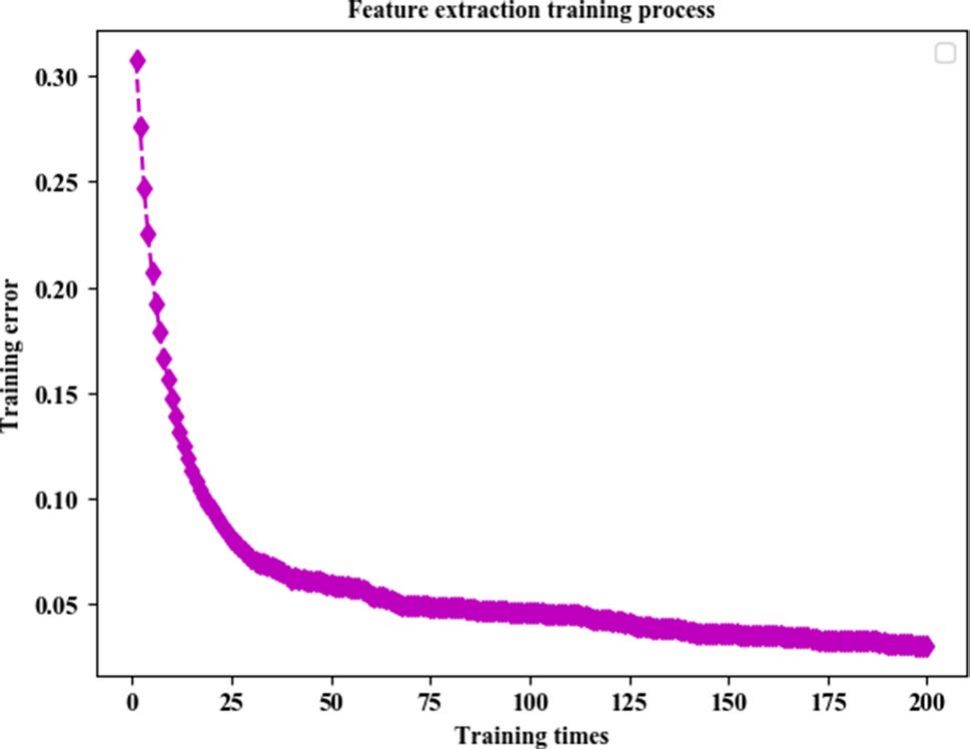
Training process of feature extraction model based on Autoencoder.

In this paper, 70,000 pieces of training data are used to train the feature extraction model based on the Autoencoder. The training process is shown in Fig. 1. In the figure, the horizontal axis of the coordinate system represents the number of trainings of the feature extraction model, and the vertical axis of the coordinate system represents the training error of the model. The initial training error of the feature extraction model based on the self-encoder is 0.308, and the training error tends to converge during the 126th training, and finally stabilizes at about 0.04.

### Predictive model training

**Figure 2:**
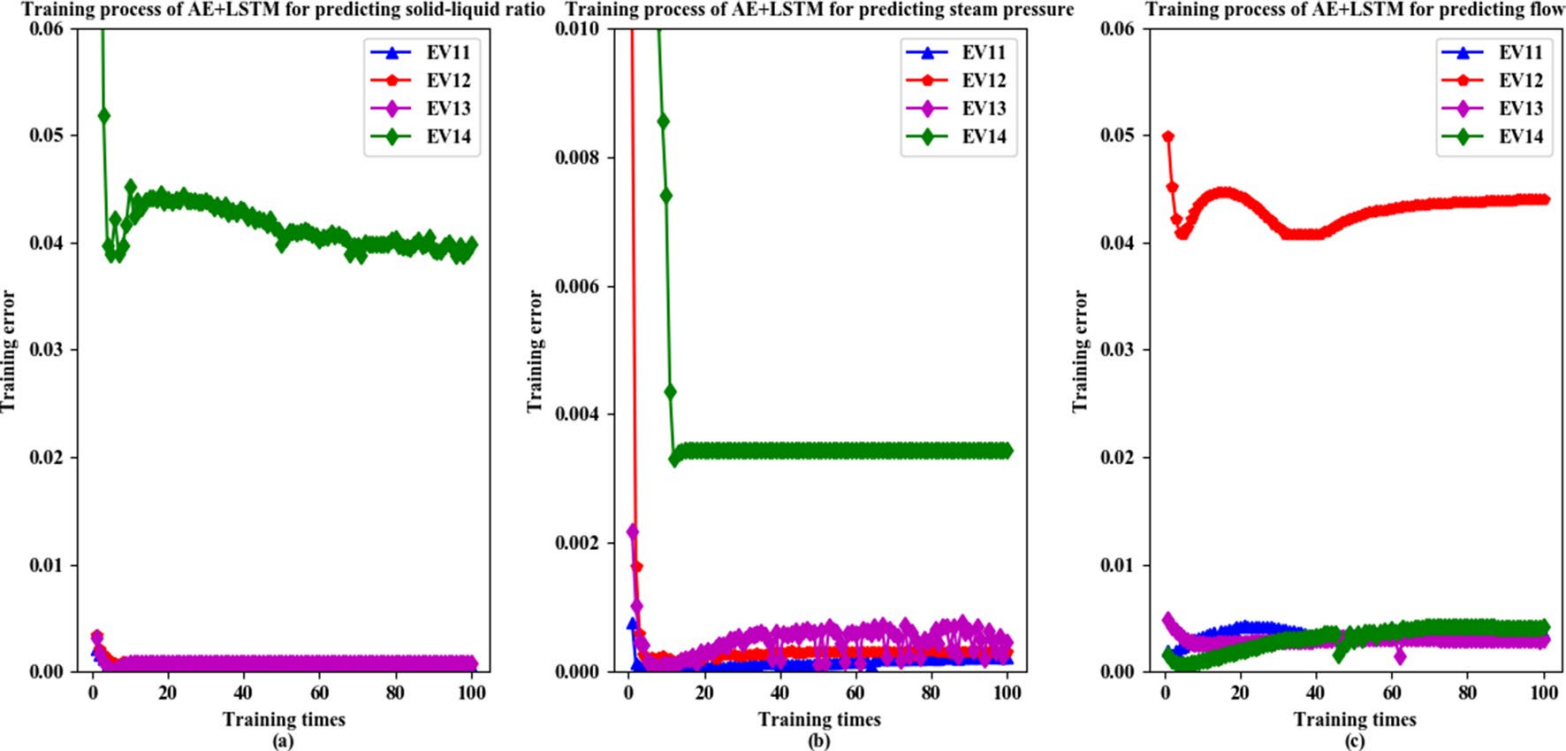
Training process of DBN+LSTM model.

In this paper, a trained feature extraction model based on Autoencoder is used to extract 70,000 pieces of training data, and the formed feature data set is input into the LSTM for training. In the training of the LSTM, this paper takes the solid–liquid ratio of EV11 to 14 evaporation tank, steam pressure and salt leg flow rate as the prediction targets for the model training. The training process is shown in Fig. 2. The horizontal axis denotes the number of training times, and the vertical axis is the training error of each training of the model.

Figure 2a shows the training process of training the AE+LSTM model with EV11 to EV14 solid–liquid ratio as the prediction target. The blue line indicates the training process with EV11 solid–liquid ratio as the prediction target. The initial training error of the model is 0.0021. The model tends to converge at the 64th training and eventually stabilizes at around 0.00038. The red line indicates the training process with EV12 solid–liquid ratio as the prediction target. The initial training error of the model is 0.00349. The model tends to converge at the 15th training and stabilizes at around 0.0006388. The purple line indicates the training process with EV13 solid–liquid ratio as the prediction target. The initial training error of the model is 0.0032, and the model tends to converge at the 13th training, which is stable at around 0.000792. The green line indicates the training process with EV14 solid–liquid ratio as the prediction target. The initial training error of the model is 0.106, and the model tends to converge at the 14th training, which is stable at around 0.044.

Figure 2b shows the training process of training the AE+LSTM model with EV11 to EV14 vapor pressure as the prediction target. The blue line indicates the training process with EV11 steam pressure as the prediction target. The initial training error of the model is 0.00076. The model tends to converge during the 34th training and eventually stabilizes at around 0.00033. The red line indicates the training process with EV13 steam pressure as the prediction target. The initial training error of the model is 0.01. The model tends to converge during the 15th training and eventually stabilizes at around 0.0002. The purple line indicates the training process with EV13 steam pressure as the prediction target. The initial training error of the model is 0.00219, and the model fluctuates around 0.0005. The green line indicates the training process with EV14 steam pressure as the prediction target. The initial training error of the model is 0.0111. The model tends to converge during the 13th training and eventually stabilizes around 0.0034.

**Figure 3:**
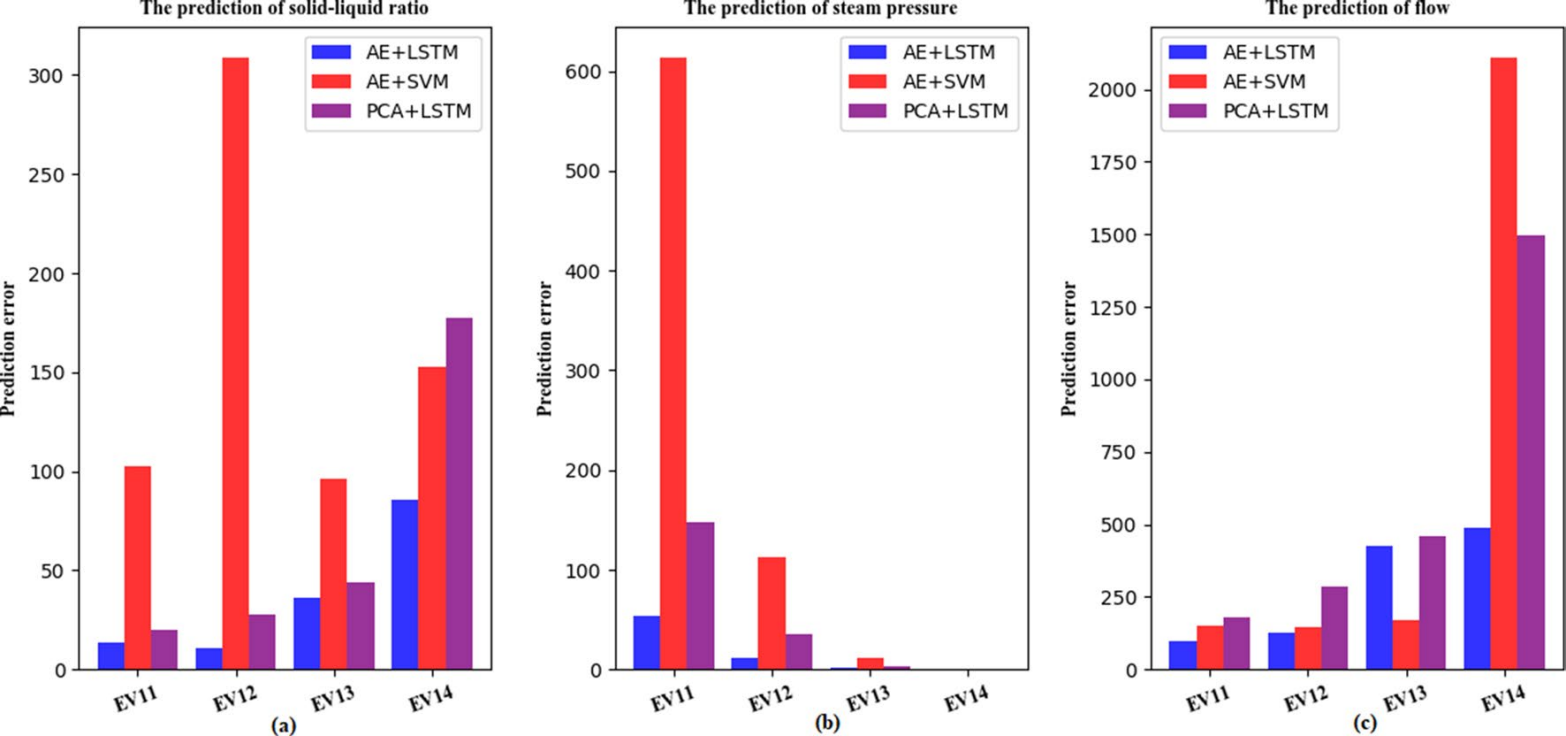
Forecast comparison of AE+LSTM model.

Figure 2c shows the training process of training the AE+LSTM model with EV11 to EV14 salt leg flow as the prediction target. The blue line represents the training process with EV11 salt leg flow as the prediction target. The initial training error of the model is 0.002. The model tends to converge during the 41st training and eventually stabilizes at around 0.0034. The red line represents the training process with EV11 salt leg flow as the prediction target. The initial training error of the model is 0.05. The model tends to converge during the 47th training and eventually stabilizes at around 0.042. The purple line indicates the training process with EV11 salt leg flow as the prediction target. The initial training error of the model is 0.005. The model tends to converge during the 20th training and eventually stabilizes at around 0.0027. The green line represents the training process with EV11 salt leg flow as the prediction target. The initial training error of the model is 0.0016. The model tends to converge during the 71st training and eventually stabilizes at around 0.0042.

### Predictive analysis

After the AE+LSTM model training is completed, this article uses 10,000 pieces of test data to evaluate its prediction effect. In this paper, the solid–liquid ratio, steam pressure and salt leg flow rate of the EV11 to 14 evaporation tanks are used as the prediction targets. PCA+LSTM(i.e., principal components analysis combine with Long short-term memory) and AE+SVM(i.e., Autoencoder combine with Support Vector Machine) models are compared for prediction, as shown in Fig. 3. The PCA+LSTM model considers the time-series information of the data, but because its feature extraction model is PCA, the extracted features are fixed. The AE+SVM model uses AE as the feature extraction model, thus the extracted features are continuously learned, but the SVM model does not consider the temporal information of the data. Figure 3a uses solid–liquid ratio as the target for prediction. From EV11 to EV14, the AE+LSTM model achieve the minimum prediction error. However, AE+SVM model present the worst performance. the prediction error of PCA+LSTM is higher than AE+LSTM. The solid–liquid ratio is a parameter with large fluctuations in the multi-effect evaporation salt production process, we can conclude in this experiment that considering the temporal information will get a better effect and the performance of AE+LSTM is better than PCA+LSTM. Figure 3b is based on the prediction of steam pressure. The AE+LSTM model has the best effect and the smallest prediction error, followed by PCA+LSTM. The changes in the vapor pressure of EV13 and 14 evaporation tanks are generally stable, but the performance of AE+SVM is very bad. AE+LSTM and PCA+LSTM model all consider the time information of data, we conclude that the time information of data is very importance to the data of multi-effect evaporation salt production process. Figure  3c is based on the prediction of the salt leg flow rate. The AE+LSTM model has the best effect and the smallest prediction error. The salt leg flow rate is a parameter with large fluctuations. AE+LSTM not only considers the changes in data characteristics, but also considers the time series information of the data. Therefore, the AE+LSTM proposed in this paper can better predict the multi-effect evaporation salt production data.

## Discussion

### Feature extraction

Nowadays, computer technology has been integrated into all walks of life. With the improvement of computer storage performance, more and more data can be recorded, and the data dimension is developing toward a high dimension. With the continuous growth of data dimensions, “dimensional disaster” has gradually become a concern for researchers in the computer field, because too high data dimensions will make the performance of various learning models poor. Therefore, in the face of the challenges brought by high-dimensional data, feature extraction techniques emerged at the historic moment^11^. Feature extraction is mainly accomplished by transforming the feature space^12^, which is an important technique for representing high-dimensional data, and is a necessary preprocessing step for large-scale industrial data^13,14^. It is used in pattern recognition, data mining, and computer vision. All fields have applications^15^. In detail, feature extraction is to find the low-dimensional feature subspace of the data through mathematical methods^16^, and map the high-dimensional data into its low-dimensional subspace, so that the original data can be well represented in the low-dimensional subspace and distribution^17,18^. Different feature extraction methods have different performances^19,20^. Typical feature extraction methods include principal component analysis (PCA).

Principal components analysis (PCA) is a typical feature extraction algorithm, widely used in the field of data compression and data analysis. PCA projects the high-dimensional features of the original data into a low-dimensional subspace in a linear or non-linear manner according to mathematical theory, and this low-dimensional subspace retains the original data space distribution as much as possible. In the projection process, PCA can identify the direction vectors called principal components from the data. In these direction vectors, the data in the original data set have the largest change in data value, that is, the data in the original data set. The changes are mainly reflected in these main direction vectors, thus the original data can be well represented using these direction vectors.

PCA has good theoretical properties and attracts a large number of researchers. The classic PCA algorithm is widely used in the field of feature extraction. Not only that, many improved algorithms based on PCA have been proposed to better solve the corresponding problems. In order to be able to extract more useful features, Yi et al. proposed a new PCA algorithm, namely the Joint Sparse Principal Component Analysis (JSPCA) algorithm^21^. The JSPCA algorithm relaxes the orthogonal constraints of the transformation matrix so that more features can be freely combined to represent the data in low dimensions. JSPCA imposes a joint sparse constraint on the objective function, that is, a paradigm constraint on the loss term and the regular term, which improves the algorithm’s greatness. The author analyzes the theory of the algorithm and gives a simple and effective optimization plan. Experiments show that this algorithm can better extract useful features in the data set. By improving the PCA algorithm, Fan et al. proposed a learning framework based on multiple similarity metric subspaces, namely an improved principal component analysis (MPCA) algorithm^22^. MPCA calculates three similarity matrices according to the similarity measurement method: interactive information matrix, angle information matrix, and Gaussian kernel similarity matrix. The author uses the feature vector of the similarity matrix to generate a new subspace, that is, the similarity subspace, and finally uses the feature selection method to generate a new complete similarity subspace, so as to realize the feature extraction of the data. In the process of data analysis, outliers are a problem that cannot be ignored. Thus Rahmani et al. proposed a simple, powerful, and robust principal component analysis algorithm^23^. In the article, the author proposes that as long as there are enough data points in the low-dimensional subspace, then these points have a strong correlation. In contrast, outliers usually do not exist in low-dimensional structures, thus outliers are unlikely to have a strong similarity to a large number of data points. As a result, outliers can be distinguished. This algorithm calculates the data correlation by normalizing the Gram matrix of the data, and then recovers the subspace through a small number of data points. Because its calculation process only involves matrix multiplication, this method is faster than the classic PCA calculation, and it can still perform well in the presence of abnormal points in the data.

#### LSTM neural network prediction model

**Figure 4:**
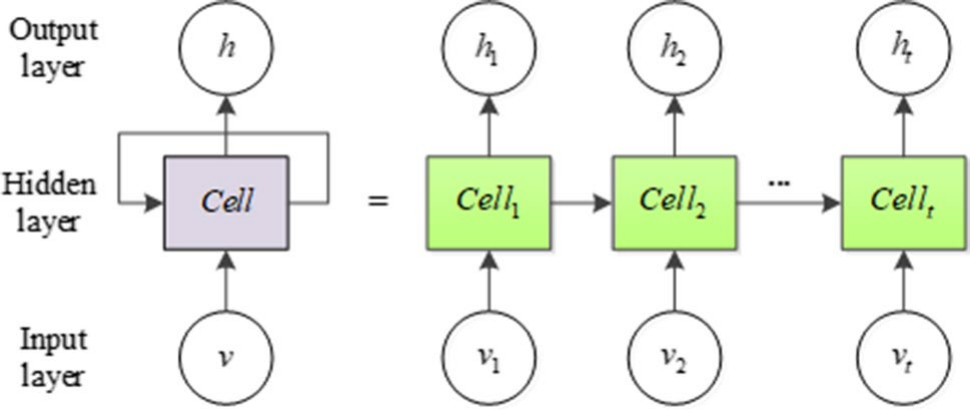
The schematic diagram of recurrent neural network.

Long short-term memory (LSTM) model is a kind of recurrent neural network. It is proposed to solve the long-term dependence of RNN. Due to its unique design, it is suitable for predicting events with long-time intervals in time series. LSTM has the characteristics of recurrent neural network, as shown in Fig. 4.

LSTM is composed of input layer, hidden layer and output layer. The state of the hidden layer neuron module at the current time point will affect the state of the module at a later time point. Time-series data will affect each other, that is, there is a causal relationship between the data at the current time point and the data at the previous time point and after the time point. The recurrent neural network can learn the association between the time-series data through the structure shown in Fig. 1. Therefore, the effect of the prediction of time-series data is outstanding. However, the traditional recurrent neural network can only remember the correlation between the data in a short period of time. To solve this problem, LSTM, because of its special structural design, can Learn through association, thus it has a good performance in the prediction of time series data. LSTM model is a special improvement based on RNN, which can be used to learn the long-term dependence of the data^24^. Illumination and photovoltaic data are typical time-series data. In order to accurately predict the photovoltaic power in the smart grid, Abdel-Nasser et al. used LSTM to predict the output power of the photovoltaic system, thereby providing an important guarantee for the safe operation of photovoltaics^25^. Qing et al. proposed a novel illumination forecasting scheme to predict solar irradiance through LSTM^26^. Video data is a commonly used time-series data in computer vision. In the study of picture subtitles, automatic description of natural language based on video content has attracted widespread attention. Gao et al. proposed a novel LSTM-based framework that can convert video into natural sentences. The framework integrates the attention mechanism with LSTM to capture the salient structure of the video and explore the correlation between multi-modal representations to generate sentences with rich semantic content^27^. In the 3D skeleton sequence, human motion recognition has attracted the attention of many researchers. LSTM has advantages in modeling dependencies. Liu et al. proposed an LSTM framework, that is, global context awareness attention LSTM, used for bone motion recognition. The algorithm can selectively focus on information-rich joints, and further improve the attention ability in each frame by using the global context storage unit^28^. Weather data is also a typical time-series data. Huang and others have developed a framework based on convolutional neural networks and LSTM to solve the problem of air pollutant index prediction, and use historical data to predict the air pollution index^29^. Because of its structural design, LSTM has very good performance in time-series data learning. When learning time-series data, LSTM learns the current and past states of neurons to generate potential characteristics of time series data, thus it can be used for the prediction of salt production data in the multi-effect evaporation salt production process.

#### Autoencoder

Autoencoder (AE) can learn artificial neural networks from the data through unsupervised learning. The AE is composed of an encoder module and a decoder module. The encoder maps the data to the feature subspace, and the decoder is responsible for reconstructing the data through the features. Chorowski et al. applied self-coding neural networks to speech waveforms^30^, and used Autoencoder neural networks to extract meaningful potential speech features in an unsupervised manner. The goal of this algorithm is to learn the features of higher-order semantic content from the signal. Because the learning behavior of the self-encoder model depends on the representation of potential constraints, the author applies three variants of the self-encoder and different constraints so that the model can learn the features that meet the needs. Zeng et al. proposed a hybrid model combining a stacked self-encoder and Mel frequency cepstrum coefficients^31^, which extracts key information through the self-encoder to improve model performance. The features involved in image processing are high-dimensional. For systems like facial expression recognition, selecting the most important features is a very critical task. Usman et al. studied the performance of deep self-encoders in feature extraction^32^, performing facial expression recognition on multiple hidden layers.

Compared with other feature selection and size reduction techniques, feature performance extracted from stacked self-encoders behaves better. Condition monitoring is one of the main tasks in the industrial process. Mechanical parts such as motors, gears and bearings are the main components of the industrial process. Any failure in them may cause the entire process to stop completely, resulting in serious losses. Therefore, it is critical to predict defects before they occur, but most methods are based on the processing of raw sensor data, which is complicated and inefficient. The latest development of feature extraction methods based on self-encoders provides methods for them, but they are mainly limited to the field of image and audio processing. Based on self-encoders and online sequential learning networks, Roy et al. developed an automatic feature extraction method for online status monitoring^33^. Experiments show that the method performs well. Effective condition monitoring can improve the reliability and safety of equipment. Feature extraction determines the performance of the monitoring model to diagnose faults. Maurya et al. proposed a feature extraction technology based on the fusion of low-order features and high-order features^34^ to detect machines Failures and potential anomalies. The author uses signal processing techniques to extract low-order data features, and uses deep neural networks based on stacked Autoencoders to extract high-order data features. The acoustic data set collected by the air compressor verifies the effectiveness of the proposed method.

On the feature extraction problem, how to map the data into the appropriate subspace is the key to the problem, and the dimensions of the original data also affect the performance of the feature extraction algorithm. High-dimensional data will greatly reduce the performance of the classic feature extraction algorithm, making it impossible to extract more important features in the data. The feature extraction algorithm based on neural network, because of its structural design, can be reflected in the feature extraction of high-dimensional data with very good performance.

**Figure 5:**
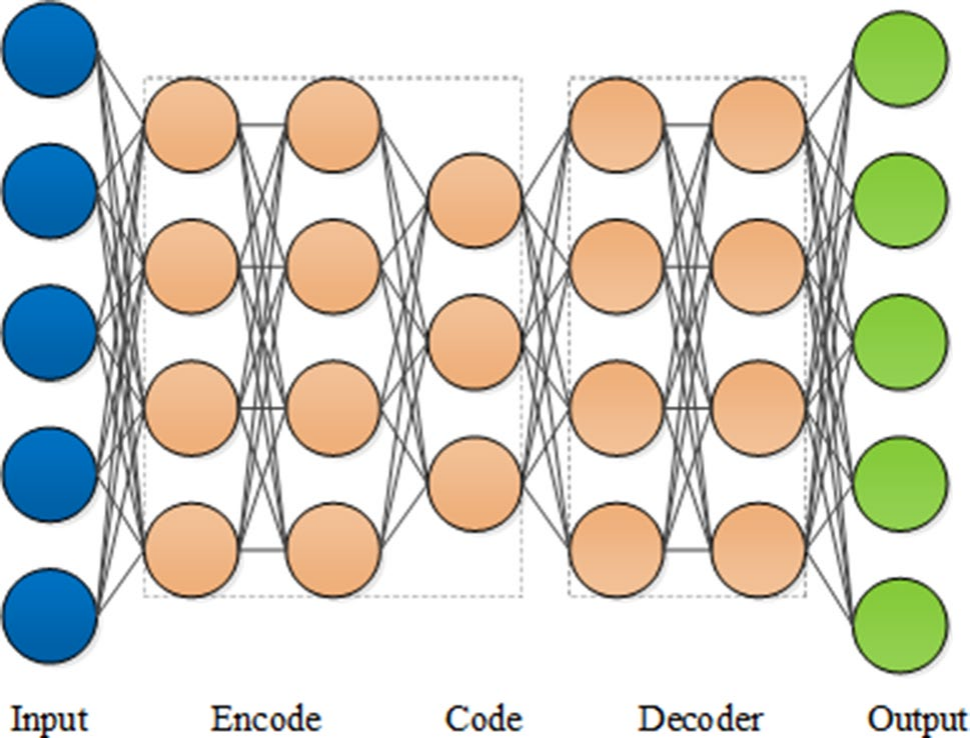
The schematic diagram of Autoencoder.

The Autoencoder is composed of an input layer, a hidden layer, and an output layer, and its structure is shown in Fig. 5, where the hidden layer is divided into an encoder and a decoder according to functions. Autoencoder and deep-confidence network have different training methods. Deep-confidence network trains RBM layer by layer, while Autoencoder trains the entire network. In the training process, the data enter the network from the input layer of the autoencoder, and the features of the data are learned by the encoder, and the learned features are sent to the decoder for decoding, that is, the original data are reconstructed by the learned features. In this process, the function of the hidden layer in the center of the Autoencoder is to obtain the characteristics of the data. The Autoencoder adjusts the entire network through reconstruction errors, and this process is repeated until the reconstruction error values tend to converge. The trained Autoencoder can be used for feature extraction, but it does not use the entire network for feature extraction. The Autoencoder only uses the parameters of the input layer and the encoder in feature extraction.

In traditional Autoencoder, the activation functions used by hidden layer neurons are mostly sigmoid activation functions. This type of activation function maps the data to a low-dimensional subspace through a nonlinear transformation. The purpose of the entire mapping is to make the data features as separable as possible. In the study of multi-effect evaporative salt production data, the extracted features need to be as separable as possible, but the individual features conform to the normal distribution as much as possible, which is conducive to the prediction of the prediction model later. Therefore, this paper makes some improvements on the autoencoder model.

## Methods

This article mainly studies how to use the Autoencoder for feature extraction of multi-effect evaporation salt production data. From the analysis of the aforementioned Autoencoder model, it is known that the Autoencoder is composed of an encoder and a decoder. The activation function does not make the entire model well learn the characteristics of multi-effect evaporation salt production data. Therefore, this paper proposes a feature extraction algorithm based on Autoencoder for the feature extraction of multi-effect evaporation salt production data as shown in Table 2.

**Table 2:**
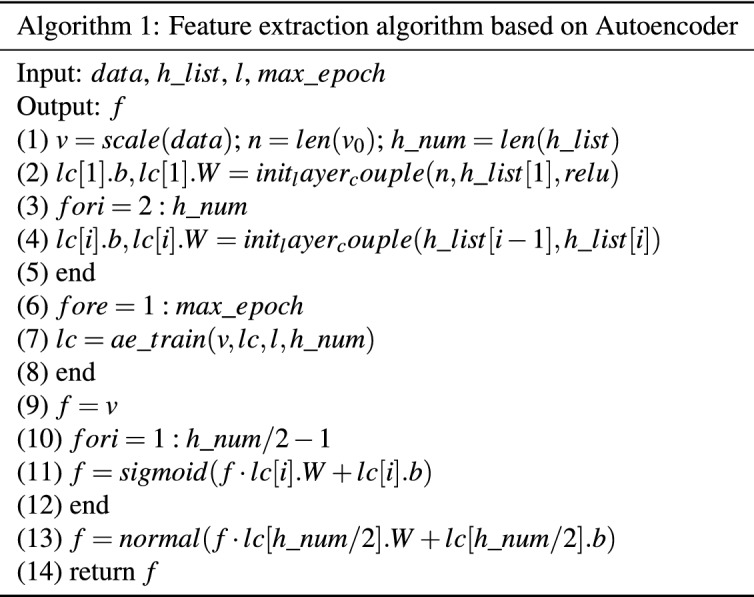
Feature extraction algorithm based on Autoencoder.

Algorithm 1 is a feature extraction algorithm based on the Autoencoder. This algorithm has improved the multi-effect evaporation salt production data, mainly to make changes in the activation function of the encoder. The activation function uses the sigmoid function, as shown in formula (2), but the hidden layer that plays the coding function uses the normal distribution function as the activation function, as shown in formula (3).

2 $$\begin{aligned} sigmoid(x)= & \, \frac{1}{1+e^{-x}} \end{aligned}$$

3 $$\begin{aligned} f(x)= & \, \frac{1}{\sqrt{2\pi }\sigma }e^{-\frac{(x-\mu )^2}{2\sigma ^2}} \end{aligned}$$

In Algorithm 1, the input of the algorithm is the salt-making data, a list of the number of neurons included in each hidden layer of the autoencoder $h\_list$ , the learning rate l and the number of training times $max\_epoch$. Step (1) normalize the salt production data to obtain the standardized data *v*, and obtain the data dimension and the number of hidden layers $h\_num$ through the len function. Steps (2) to (5) are the initialization of each hidden layer of the Autoencoder. The init_layer_couple function initializes the neurons of the adjacent layer. *Lc*[1] is the subnetwork composed of the display layer and the first hidden layer, *lc*[1].*b* is the hidden layer bias of the subnetwork, *lc*[1] .*W* is the weight matrix of the self-network. Steps (6) to (8) are the training process of the Autoencoder. The specific training process is shown in Algorithm 2. Algorithm 2 is the training process of the Autoencoder, as shown in Table 2. The input of the algorithm is data v, the subnet list *lc* of the Autoencoder, the learning rate l, and the number of subnets $h\_num$. Steps (2) to (6) are the forward propagation process of the encoder. Here, we set the activation function of the neuron at the end of the encoder to be a normal distribution function, and the activation function of the remaining hidden layers is the ReLU function. Steps (7) to (8) are used to calculate the gradient of the network, and Steps (9) to (12) are used to update the parameters in the network. The algorithm finally returns the updated *lc* list. Steps (9) to (13) of Algorithm 1 are the process of acquiring the data features of the Autoencoder. Through the above steps, the Autoencoder has been completely trained. Finally, the trained Autoencoder coding layer is used for data feature extraction. Finally, the algorithm returns the extracted data features. In this paper, Algorithm 1 and LSTM are combined to form the AE+LSTM prediction algorithm to predict the multi-effect evaporation salt production data.

Algorithm 2 is the training process of the Autoencoder, as shown in Table 3. The input of the algorithm is data *v*, the subnet list *lc* of the Autoencoder, the learning rate *l*, and the number of subnets $h\_num$. Steps (2) to (6) are the forward propagation process of the encoder. Here, we set the activation function of the neuron at the end of the encoder to be a normal distribution function, and the activation function of the remaining hidden layers is the ReLU function. Steps (7) to (8) are used to calculate the gradient of the network, and Steps (9) to (12) are used to update the parameters in the network. The algorithm finally returns the updated *lc* list. Steps (9) to (13) of Algorithm 1 are the process of acquiring the data features of the Autoencoder. Through the above steps, the Autoencoder has been completely trained. Finally, the trained Autoencoder coding layer is used for data feature extraction. Finally, the algorithm returns the extracted data features. In this paper, Algorithm 1 and LSTM are combined to form the AE+LSTM prediction algorithm to predict the multi-effect evaporation salt production data. After that, the features are input into the LSTM for training and prediction.

**Table 3:**
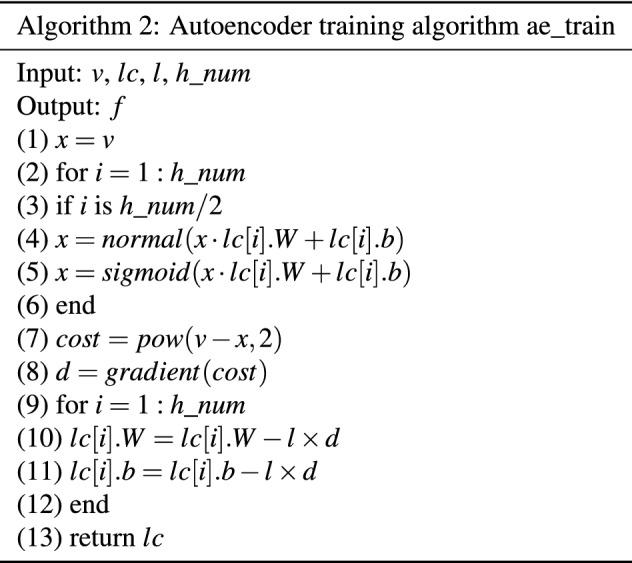
Autoencoder training algorithm ae_train.

**Figure 6:**
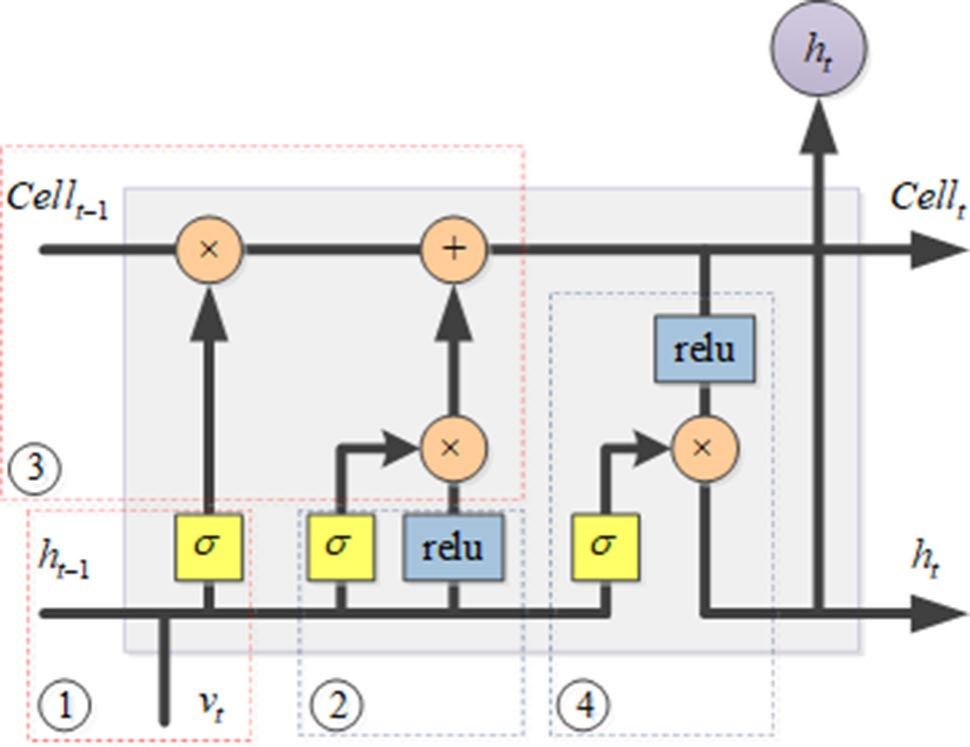
The schematic diagram of hidden layer neuron structure of LSTM.

LSTM has a special design for long-term memory on its hidden layer neurons, so that the entire network can learn long-term associations between data. Its hidden layer neuron structure is shown in Fig. 6. In Fig. 6, the hidden layer neuron of LSTM has three kinds of inputs, namely the input of the hidden layer neuron at time t, the output of the hidden layer neuron $h_{t-1}$ at time $t-1$ and the state parameter of the hidden layer neuron $Cell_{t-1}$ at time $t-1$. The LSTM hidden layer neuron is mainly composed of four modules. In module 1, the function $\sigma$ of the function is to selectively forget some information in the hidden layer neuron $h_{t-1}$ output at time $t-1$ and the input data $v_t$ at time *t*. The output of module 1 is shown in formula (4).

4 $$\begin{aligned} s_{t}=\sigma (W_{s}[h_{t-1},v_{t}]+b_{s}) \end{aligned}$$

In module 2, the role of this module is to update information, and selectively update the information in the hidden layer neuron output $h_{t-1}$ at time $t-1$ and the input data $v_t$ at time *t*. The output of module 2 is shown in formulas (5) and (6).

5 $$\begin{aligned} i_{t}= & \, \sigma (W_{i}[h_{t-1},v_{t}]+b_{i}) \end{aligned}$$

6 $$\begin{aligned} C_{t}= & \, \sigma (W_{c}[h_{t-1},v_{t}]+b_{c}) \end{aligned}$$

The function of module 3 is to update the state of the hidden layer neurons at the current moment according to the outputs of module 1 and module 2. The update formula is shown in formula (7).

7 $$\begin{aligned} Cell_{t}=s_{t}\times Cell_{t-1}+i_{t}\times C_{t} \end{aligned}$$

After the update operation of module 3, the state of the hidden layer neurons is updated. Module 4 extracts the features of the hidden layer neuron output $h_{t-1}$ at time $t-1$ and the input data $v_t$ at time *t* through the current state of the neuron, and outputs the current state hidden layer, as shown in formulas (8) and (9).

8 $$\begin{aligned} o_{t}= & \, \sigma (W_{o}[h_{t-1},v_{t}]+b_{o})\end{aligned}$$

9 $$\begin{aligned} h_{t}= & \, o_{t}\times relu(Cell_{t}) \end{aligned}$$

Through the special design of the above process, LSTM can remember and forget the features in the data, and iterate the state of the hidden layer neurons at different times to learn the long-term association of the data. Therefore, LSTM can perform learning tasks on time-series data, especially the data that have long-term dependence in the data, and obtain better learning results. This article considers the time series of multi-effect evaporation salt production, and in the multi-effect evaporation salt production process, the parameter state at the current time point will affect the parameter state at the future time point, and the effect of the reaction in the multi-effect evaporation tank will continue very long. Long time, that is, there is a long-term dependence between data. Therefore, this paper uses LSTM as the prediction model to predict the multi-effect evaporation salt production data.
